# Modelling the Balance Axiom in Flow Theory: A Physiological and Computational Approach in STEAM Education

**DOI:** 10.3390/s26010038

**Published:** 2025-12-20

**Authors:** David Antonio Rosas, Natalia Padilla-Zea, Daniel Burgos

**Affiliations:** 1 GUMTS Research Group on Fashion, Technology, and Sustainability, Universidad de Diseño, Innovación y Tecnología (UDIT), Av. Alfonso XIII, 97, 28016 Madrid, Comunidad de Madrid, Spain; david.rosas@udit.es; 2 Research Institute for Innovation & Technology in Education (UNIR iTED), Universidad Internacional de La Rioja (UNIR), Av. de la Paz, 137, 26006 Logroño, La Rioja, Spain; daniel.burgos@unir.net; 3 MIU City University Miami, 141 NE 3rd Ave, Suite 700, Miami, FL 33132, USA

**Keywords:** balance, flow theory, physiology, STEAM

## Abstract

This paper addresses the axiom of balance in Flow Theory from a physiological-and-group-based approach by a quasi-experimental study using mixed methods across two action–research cycles, each divided into pre-test, intervention, and post-test phases. The study involved 56 students in two control and two experimental groups attending robotics and design STEAM courses in natural settings, wearing Polar H10 bands. Each group participated in nine one-hour sessions, with the same instructor. While flow in control groups was measured with intuition-based teacher actions, in experimental groups the teacher received support from a synchronous physiological flow advisory system. Data from these groups were analysed using nonlinear techniques, finding preliminary evidence that suggests (1) more quickly reaching of the Zone of Proximal Development when the teacher has physiological guidance, (2) mathematical physiologically-based support for the axiom of balance of Flow Theory, and (3) nonlinear analysis in group contexts offer quantification to the previously found contradictions in Flow Theory. Moreover, these findings propose new hypotheses and potential redefinitions in Flow Theory.

## 1. Introduction

Flow Theory [1] is a motivational theory developed from its earliest stages within a phenomenological paradigm [2]. Because of this, from its very roots, research on flow has focused on individuals’ subjective and conscious experiences while engaging in an activity [1], in contrast to laboratory settings that lack ecological validity [2]. Precisely because it deals with subjective and volatile phenomena, it has relied on concepts that are difficult to operationalize, both individually and at the group level [3,4,5,6,7].

Moreover, Flow Theory explains why individuals persist in participating in activities due to the subjective combination of joy and performance achieved (optimal experience), making the tasks self-rewarding [1,2,3]. Core flow experience phenomenology is multifaced, including high concentration, sense of control, loss of self-awareness, and distortion of temporal sense [3]. Moreover, optimal experiences are characterized by the development of activities in a fluid manner, without apparent exertion, that gives name to the theory [1,2,3,4].

Since classical studies by [3], flow experience has been characterized by nine dimensions, including balance between challenges and skills, in which individuals perceive their abilities to match the demands of the activity; merging of action and awareness; and the presence of clear goals. Flow is also characterized by unambiguous feedback, as well as by intense concentration in the task at hand, alongside a loss of self-consciousness. Moreover, optimal experiences involve a transformation of time, in which temporal perception becomes distorted. Finally, the activity is undertaken for its own sake, being self-rewarding (autotelic nature).

Additionally, several preconditions are necessary to achieve individual and collective flow (see [3,5]), a key point addressed in this paper. One of the first points to be identified is the balance between subjective skills and the perceived challenges of a task, which is considered one of the main axioms of Flow Theory [1,3,5]. This principle establishes that a precondition for an individual to achieve the Flow State is a perceived balance between the person’s subjective skills and the perceived task’s challenges. In this regard, in a systematic review, the authors of [5] found balance-related dimensions in 13 out of 34 validated questionnaires assessing flow dimensions as preconditions to achieve Flow.

Nevertheless, the concept of skills, challenge, and balance evolved since the early works of Csikszentmihalyi [1], as they are not easy to define and measure in an operational manner. Furthermore, in [8], skills are defined as perceived competencies in a particular task, while challenges refer to the demands associated with that activity. Balance is understood as the perceived difficulty in relation to other activities. Importantly, all these components are moderated by perceived importance and achievement motivation, which may be socially mediated.

The first model of Flow focused on the axiom of balance shown in Csikszentmihalyi [1], p. 49. This model features, on the x-axis, action capabilities (skills) and, on the y-axis, action opportunities (challenges), along with a 45-degree sloped band called as the Flow Channel. Additionally, two other triangular zones represent the emotional states antagonistic to Flow: anxiety above the channel and boredom below it [3], p. 259. The model introduces the idea of balance between skills and challenges as a predictor of the Flow state [1]. In this context, when there is a balance between skills and challenges, the individual is in a state of Flow; if the challenges exceed their capabilities, they will feel anxiety; whereas, if the challenges are too easy for their skills, they will end up feeling bored [1,3]. Similarly, Csikszentmihalyi [3], p. 261 considers it a “diachronic composite model illustrative of how flow occurs over time”. Therefore, by sequentially connecting experimental points that represent a time series of measurements on the graph, the concept of Flow-paths is reached, aimed to assess associations between the patterns described by the Flow-paths and educational performance [9]. However, the channel model, while elegant, may present issues. For instance, Csikszentmihalyi and Csikszentmihalyi [3], pp. 255–260 provide two examples of Flow Questionnaires from an Experience Sample Method study, but the model’s predictions about emotional states might not align with users’ reports: a data point in the channel model suggesting boredom based on skills and challenges questions was instead described by the respondent as feeling excited; conversely, a data point suggesting anxiety was reported by another respondent as slightly boring. Therefore, the theoretical predictions about emotional states in the model may not match those reported by the users. Moreover, Shin [10] found that correlation between the balance variable and Flow state is statistically significant but weak (r = 0.206, *p* < 0.01). Likewise, Pearce, Ainley and Howard [9] mathematically defined the balance between skills and challenges using the expression (1):

Challenge/Skills = 1(1)

The use of a linear relationship, as derived from Equation (1), may simplify the representation of the phenomenon, which is described as inherently subjective, volatile, and nonlinear [1,9]. This mismatch between the model’s structure and the phenomenon’s nature may undermine its validity from a computational perspective.

Massimini and Carli [11] improved the previous model by normalizing the skills and challenge parameters as Z values. This requires a series of questionnaires, where each individual response is adjusted by subtracting the series’ mean and dividing by its standard deviation. This resulted in the four-quadrant model [12]. In this model, two orthogonal axes represent skills and challenges as Z values, and the four defined quadrants predict emotional states clockwise: flow, boredom, apathy, and anxiety. Flow occurs only when both skills and challenges are above the weekly mean and they are balanced [11], as opposed to three other antagonistic emotional states. Moneta and Csikszentmihalyi [12] concluded that the balance between challenges and skills, independently considered, improve the quality of an experience, provided that concentration, desire to perform the activity, involvement, and happiness are present as dependent variables.

Later, the Experience Fluctuation Model (EFM) (see [11,13]) was defined. This model is graphically represented by a circle divided into eight radial sections with two orthogonal axes at the centre, corresponding to normalized Z values of challenge and skills. The quadrants in clockwise order are arousal, flow, control, boredom, relaxation, apathy, worry, and anxiety. In the EFM, optimal experiences require both skill and challenge levels to be balanced and above a certain threshold [11,13]. Moreover, Novak and Hoffman [14] applied Principal Component Analysis to questionnaires containing skills and challenges dimensions, suggesting that the choice between four or eight channels may result arbitrarily since both are explained by these two factors, accounting for 89% of the variance. In addition, in [15] (p. 546), it is acknowledged that these sectors were established arbitrarily.

More recently, Løvoll and Vittersø [16], in an educational context, considering a validated flow scale and the EFM model, concluded that persistent balance in tasks can lead to boredom, which may contradict the balance axiom. Even more, ref. [4] suggests that Flow Theory has reached a crisis point, posing a need for a paradigm shift or a more precise definition of the concept.

In view of these difficulties, more recent flow studies have incorporated physiological techniques (i.e., [6]), primarily at the individual level in laboratory contexts and, to a lesser extent, in group-based settings [7], as we address in this work.

Although examples are limited, Heart Rate Variability (HRV) techniques have also been utilized in conjunction with flow questionnaires in flow research [1,17,18]. HRV parameters are measures of heartbeat nonlinear processes, being influenced by factors such as stress, emotions, and cognitive processes, offering insights into the modulation of the heart by the sympathetic and parasympathetic nervous systems [19,20,21]. However, these studies have predominantly been conducted in laboratory settings. Moreover, we build this work considering the results of [22]. They found significant correlations between the EduFlow scale [23] and its four dimensions with several HRV parameters in real classroom settings, thus providing physiological validation of this scale. This was made possible using non-invasive Polar H10 wearable devices to monitor HRV, considered the gold standard for such measurements [24], together with both synchronous and asynchronous monitoring software. Furthermore, the Flow Kurtz Scala (FKS) [24], which shares one dimension with EduFlow (see [23]), includes items that assess skills, challenges, and balance, components that may be evaluated with physiological measures.

Within this framework, this paper examines Flow in groups—an area that requires further research, see [5,7]—and explores how the balance principle operates in an educational ecological context through the innovative use of non-invasive physiological wearables, in contrast with phenomenological and questionnaires-based research alone.

In view of these aims, we address the following research questions:

RQ1: What is the degree of coherence between the flow reported by the control groups and the flow recalled by the teacher using the EduFlow and Flow Kurtz Scala?

RQ2: Is there a differential correlation between control and experimental groups among heart rate, balance, and flow?

RQ3: What are the differences in perceived group balance between experimental and control groups as measured by Poincaré plots?

## 2. Materials and Methods

This study adheres to the Declaration of Helsinki and complies with the Spanish Organic Law 3/2018 on Personal Data Protection. Ethical approval was obtained from UNIR’s Ethical Committee (approval code PI015/2022). Additionally, the Juvenile Prosecutor’s Office of Granada, Spain, was notified about the study’s characteristics, purposes, and data collection methods. Legal guardians were thoroughly informed, and informed consent was required for participation. Participants did not receive any compensation, and were made aware both orally and in writing of the possibility to drop out the research without any explanation or negative consequence. Data were collected anonymously, and group mean values were used in the analysis, eliminating the possibility of identifying any of the participants by their biometric data.

### 2.1. Research Design

This study employs a quasi-experimental design incorporating mixed methods [25]. It involves, two control groups and two experimental groups engaged in varied face-to-face science–technology–engineering–arts–mathematics (STEAM) projects [26], as opposed to more mechanical tasks conducted in laboratory settings [6,17,18]. Given that flow state is nonlinear and multifaceted [5,22,23,24], we conducted experimental research focusing on the balance dimension. This study combines nonlinear statistical techniques, non-invasive physiological measures, and validated flow questionnaires (EduFlow, FKS, and Swedish Flow Proneness Questionnaire) in natural educational settings, as suggested in [5]. In our research, the same teacher led two control groups with asynchronous wearable monitors and two experimental groups with the aid of computational wearable synchronous advice.

In the control groups, the teacher relied solely on intuition (i.e., observing if students appear engaged in activities without apparent difficulties, showing enjoyment, anxiety, or boredom…), which is also referred to as flow metacognition [27]. In the experimental groups, the teacher had the help of a synchronic tool [28], reporting about the flow state of the group.

Both control groups and experimental groups were engaged in two action–research cycles [29], which were structured into pre-test, intervention, and post-test phases.

Finally, after every action–research cycle, we performed a retrospective analysis, as well as at the end of the research.

### 2.2. Instruments

Students completed the Swedish Flow Proneness Questionnaire [30] to assess their ability to experience flow. Building on findings reported in [5], we utilized the FKS [24] and EduFlow [23] scales, which are validated questionnaires [5,22,23,24]. According to [31], both EduFlow and FKS scales share 73% of the explained variance in the control groups. Furthermore, their reliability, measured using Cronbach’s alpha, was 0.73 and 0.85, respectively. Moreover, the EduFlow scale demonstrated physiological and ecological validity in educational contexts, as its dimensions show multiple correlations and coherent agreement with more than 38 HRV parameters [22]. In consequence, EduFlow and FKS scales are suitable for assessing flow prevalence in technology-mediated, project-based teaching [2,22,23]. The FKS includes items measuring course progression (6 items), cognitive absorption (4 items), and task relevance (3 items), along with 3 questions for each of the subjective challenge (A1), skills (A2), and balance (A3) dimensions [24]. The EduFlow [23] questionnaire has 4 items for each dimension: cognitive absorption (D1), time transformation (D2), loss of self-consciousness (D3), and autotelic experience-well-being (D4). All items in these scales use a 7-point Likert scale, with increasing degrees of agreement, except for the items in dimensions A1, A2, and A3, which rely on a 9-point Likert scale.

Each participant wore TicWris Max watches as data loggers and Polar H10 sports bands, which are recognized for their precision in wearable electrocardiology [32] and suitability for research involving children [33]. We developed a Kotlin application using Android Studio (Bumblebee v.2021.1.1) to access the watch’s accelerometer, interactive screen buttons, and SD card, as well as to interface with the Polar H10 sports bands via Bluetooth through the Polar SDK v.3.3.6 [34]. We named these applications “Polar H10 SDK UNIR-iTED v4”, crediting the developers of the library.

The application was customized for each device to connect exclusively to a specific Polar H10 band via Bluetooth. Each band measures participants’ cardiac electrical potential at 130 Hz to record cardiac data at a frequency of 1 Hz, storing R-wave intervals, beats per minute, and timestamps in Universal Unix format on the watches’ SD cards. Data were downloaded to a PC after each session. We then used Kubios 3.5.0 software [35] to filter and detrend the signal, remove artifacts, and extract multiple cardiac parameters in CSV format. Kubios allows for the segmentation of recordings into 5 min intervals, aligning with the electrocardiological standards for children [36].

**Figure 1:**
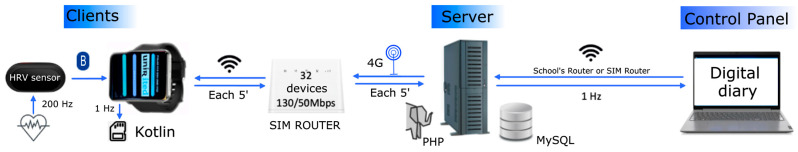
Schematic representation of EASFE.

For educational interventions, we developed two STEAM-based technological itineraries, focusing on educational robotics for 6th graders and 2D/3D graphical design for 5th graders. We graded tasks difficulty following Bloom’s taxonomy for the digital age [35]. Each instructional module is delivered in SCORM [37]. These modules were incorporated into a web application that recorded activity data at 1 Hz, including Bloom’s taxonomy classification, Unix time, and teacher activity (e.g., presenting, explaining, answering questions). It was denoted as a digital diary, being part of the teacher’s control panel (Figure 1).

With experimental groups, the teacher also used the Early Alert System of Flow Expectation (EASFE), based on the Polar H10 SDK UNIR-iTED app (v.7). Its design in depth is out of the scope of this paper, but it can be seen in [29] and will be briefly described in Section 3 (Results). The EASFE was developed using experimental data gathered from the control groups (see [29]). It relies on a mathematical classification model that identifies four flow states based on the group’s mean heart rate and its standard deviation. It also incorporates an experimentally demonstrated inverse relationship between the control groups’ heart rate, mean cognitive absorption, and mean recalled flow across 18 lessons, measured with the EduFlow scale. This system operates as a RESTful API [38], using JSON objects to communicate with PHP files on a server, interfacing with a MySQL database. Mean cardiac data are transmitted to the server every 5 min using a SIM card router, while the teacher’s control panel data logs have a granularity of 1 Hz. The router, capable of connecting up to 32 devices, provided adequate bandwidth (130/50 Mbps). Figure 1 summarizes the explained setup.

**Figure 2:**
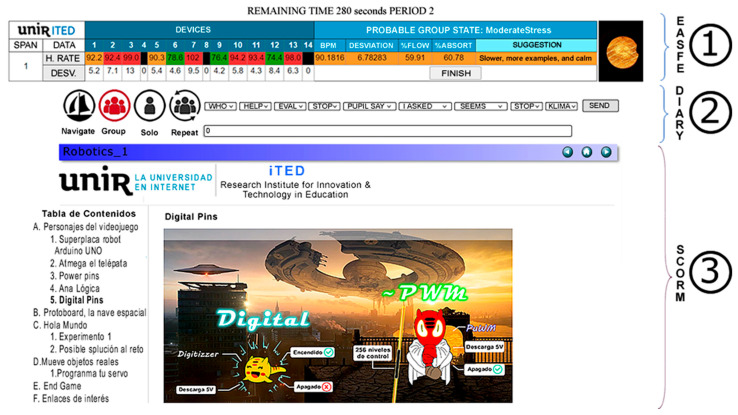
Full teacher’s control panel showing EASFE table (**1**), digital diary (**2**), and SCORM (**3**).

In the experimental groups, the EASFE information table was displayed at the top of the control panel on the classroom projector (Figure 2). This table allowed the teacher to start or stop the session by simply clicking one button. The system synchronized the smartwatch recording, divided the lessons into 5 min intervals with a countdown timer, and displayed the span number already assessed in the current period. Using a segmentation model developed from control group data and synchronous HRV parameters (heart rate and its standard deviation), the system made suggestions to improve group flow: it displayed a traffic light colour code indicating the probable group state at the end of the lesson if the suggestions were not applied. Additionally, it showed individual information for up to 14 participants, but the teacher only noted if students were absent or had turned off their smartwatches (indicated in black). Moreover, trusting the model’s early predictability, the teacher followed the same EASFE suggestions from the fifth minute until the end of the lesson, focusing on teaching.

Students also completed the same theoretical–practical exams during the pre-test and post-test stages and participated in interviews at the end.

### 2.3. Participants

In this study, two control groups and two experimental groups were involved. The control groups consisted of a 5th grade class (5N_1) and a 6th grade class (6N_1) from a primary school during the 2021–2022 academic year (Table 1). The experimental groups included students from both 5th (5N_2) and 6th (6N_2) grades of primary school during the 2022–2023 academic year (Table 1).

Additionally, all groups were led by the same teacher-researcher, a man aged between 48 and 49 years, with over 10 years of accredited teaching and research experience in primary education.

### 2.4. Experimental Procedure

We followed the research process outlined in [31], conducting a quasi-experimental research design with mixed methods involving two control and two experimental groups across two cycles of action–research [30]. Each action–research cycle comprised three stages: pre-experience, experience, and post-experience.

During the pre-experience stage, we did not identify potential non-autotelic students with the SFPQ [32]. Therefore, all students completed a practical–theoretical exam.

The experimental stage consisted of nine one-hour lesson plans designed for two STEAM project-based courses. Both courses followed a uniform structure of nine SCORM-based sessions, with materials presented via a projector and recorded in a digital diary which documented activities, time, teacher demeanour, and task complexity, based on Bloom’s taxonomy for the digital era [39].

Regarding the instructional designs, we followed the protocols described in [40]. In all courses, the first session was devoted to a general rehearsal, with instructions on the safe use of technology and becoming familiar with the research context.

The 5th-grade students first created digital comics using professional graphic design and image-editing software (7 lessons). These works, which depicted how they imagined daily life in medieval Spain, were later exhibited in a regional comic museum. After visiting the archaeological remains of the castle, the city walls, and the citadel in their town, the students then built a full-scale recreation of the site in Roblox Studio (see [41]) using its building tools without coding. They used predesigned assets, including peasants, trees, and animals to complete the scenarios (2 lessons). Due to internet access issues, Roblox Studio lessons were excluded from the experimental group dataset and only implemented in the control group, as desktop application updates failed because of firewall restrictions.

In contrast, the 6th-grade students took part in an artistic educational robotics program using mBlock5 [42], based on Scratch coding [43] and Arduino hardware [44]. They began by visiting the UNESCO Granada Geopark (Spain) and, inspired by it, they developed a simple video game with self-designed characters controlled via keyboard input, introducing them to basic coding (5 sessions). They then created small interactive sculptures resembling ethnographic objects from the region, featuring LED lights and servo-driven movements activated from their laptops (4 sessions).

In both instructional designs, artistic goals acted as creative drivers and core learning content, integrated with competencies from the Natural and Social Sciences, Technology, Engineering, and Mathematics, each addressed with different levels of depth. This may be seen as a variant of the integrated STEM (iSTEM) methodology, as discussed in [45], incorporating arts.

Students attended lessons twice weekly during school hours, with a 10 min rest period before each session. Classes were all conducted in the same ICT classroom, arranged with five parallel desk rows facing the board and clear pathways for the teacher’s movement. Each student was equipped with an identical laptop and mouse. A single teacher instructed all four groups, adopting a playful teaching approach. The teacher maintained a qualitative diary throughout the sessions, recording observations, while school-appointed tutors oversaw the lessons to ensure consistency and adherence to instructional designs. To uphold ethical standards, pretrained students independently applied HRV bands in locker rooms, ensuring proper usage via apps monitoring Polar H10 bands.

Control groups used the wearable monitoring system with data studied asynchronously to develop models, while the teacher adapted the pace of instruction flexibly based on intuitive decisions regarding group flow. Experimental groups also wore smartwatches and Polar H10 bands, but the teacher had access to the EASFE synchronous monitoring system. After the initial five minutes using the EASFE, the teacher followed system-provided advice, such as repeating explanations once or twice, and offered individual assistance or additional resources only when required. Consistency in activities and lessons across control and experimental groups was ensured by consulting digital classroom diaries, which recorded the development of instructional designs in real-time.

In the post-experience stage, we conducted student interviews. Additionally, all student groups underwent thematic exams encompassing practical and theoretical questions, like the ones presented in the pre-experience stage.

## 3. Results

**Figure 3:**
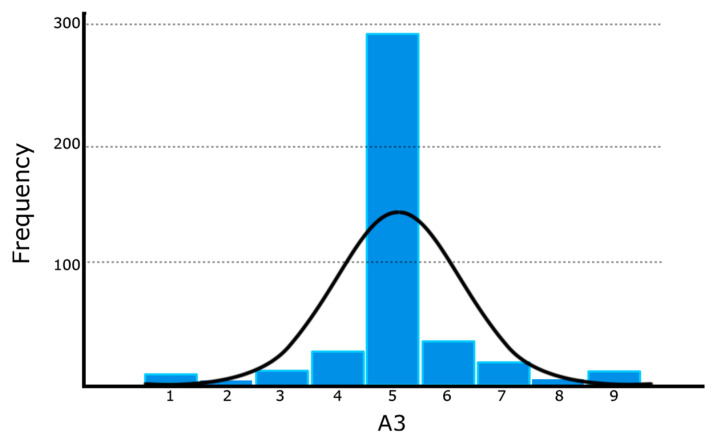
Frequency histogram of A3 dimension (balance) extracted from FKS questionnaires for all students.

Firstly, we describe results obtained with the FKS scale, concretely, with the item A3, regarding the balance of the tasks. In this context, we present the frequency histogram for this item A3 since it represents the perceived balance of the activities, based on a 9-point Likert scale (M = 5.10, SD = 1.14). The data is derived from valid responses to 407 FKS student questionnaires (Figure 3). In particular, the total number of questionnaires filled out by each group is as follows: 5N_1 submitted 101 responses, 6N_1 provided 98 answers, 5N_2 recorded 103 responses, and 6N_2 contributed 105 answers.

**Figure 4:**
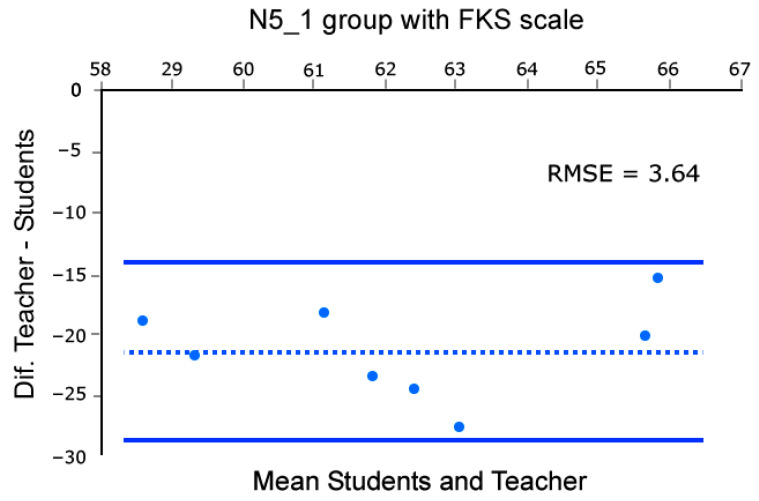
Bland–Altman [46] plot comparing teacher’s score and mean group Flow Short Scale scores for N5_1 group.

**Figure 5:**
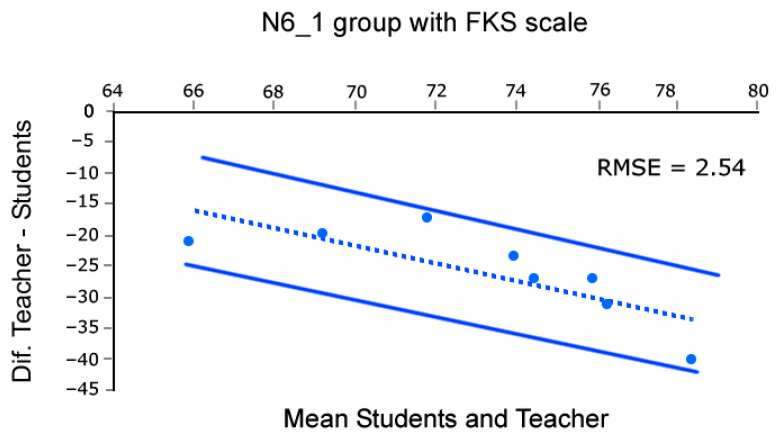
Bland–Altman [46] plot comparing teacher’s score and mean group Flow Short Scale scores for N6_1 group.

**Figure 6:**
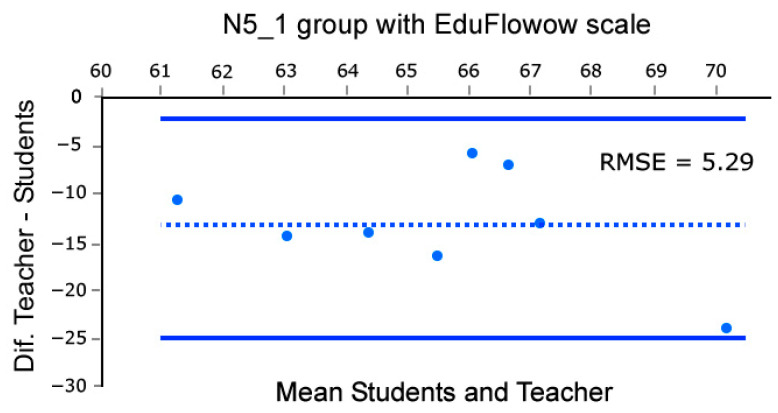
Bland–Altman [46] plot comparing teacher’s score and mean group EduFlow scores for N5_1 group.

**Figure 7:**
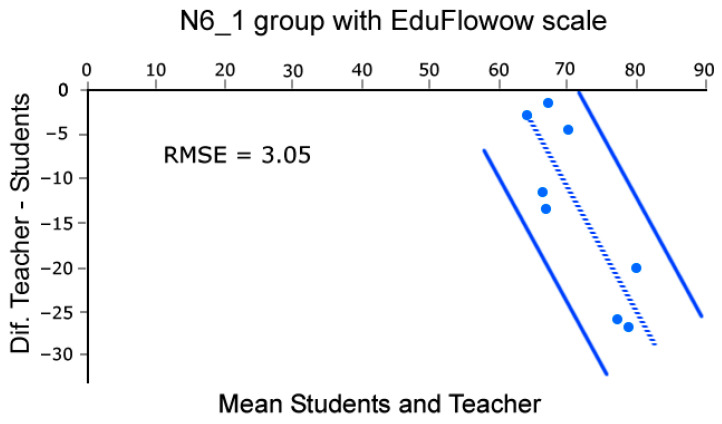
Bland–Altman [46] plot comparing teacher’s score and mean group EduFlow scores for N6_1 group.

Moreover, to assess the concordance in control groups between the mean group flow reported by students and the flow recalled by the professor using the EduFlow and FKS scales we build Bland and Altman plots [46]. This analysis was conducted across eight sessions, as the first of the nine lessons was dedicated to rehearsal and testing of the methods. Note that such analysis was conducted only in the control groups datasets, as they rely solely on the teacher’s intuition. Briefly, in Bland and Altman [46] plots, the X-axis of the scatter plot represents the average of the teacher’s flow and the students’ mean group flow, while the Y-axis represents the difference between them. This enables us to compare the coherence of datasets. Coherence is identified by distributions aligned with a balance line, with arbitrarily set confidence limits (95% in our case). To do this, we performed these calculations and plots using the BA-plotteR v.1 software [47], which can be seen in Figure 4, Figure 5, Figure 6 and Figure 7, once completed with the RMSE relative to the balance line (dashed in Figure 4, Figure 5, Figure 6 and Figure 7). In particular, Figure 4 and Figure 5 represent data obtained with FKS scale while Figure 6 and Figure 7 represent data using the EduFlow scale.

In addition, we consider the EASFE development principles [28]. EASFE was developed with mean group values from control group sessions divided in 5 min periods to make HRV parameters extracted with Kubios comparable [35]. It benefits from temporal correlation series among EduFlow scales, HR, and its standard deviation. As shown in [28], group flow reported after the first 5 min span is a good predictor to make a general recommendation for the whole lecture. Thus, during this experience, the teacher follows EASFE advice after 5 min until the end of the lecture.

Moreover, we use the previous definition of the parameter barriers in Equation (2), where challenge (A1), skills (A2), and balance (A3) are mean group values. Barriers represent perceived asperities in the ongoing tasks because A3 in control groups exhibits almost perfect balance (Figure 3), and the difference between challenge and skills ($\bar{A1}-\bar{A2}$) becomes a nonlinear measure of perceived group difficulty in tasks performed (see [28]).

(2) $$Barriers=\frac{\bar{A1}-\bar{A2}}{\bar{A3}}$$

**Figure 8:**
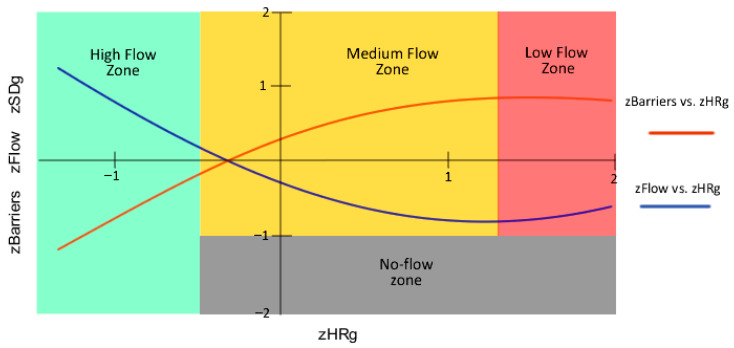
Graphical relationships between flow and barriers, shown as z values following [28]. Flow and barriers form symmetric convex–concave curves with respect to mean heart rate (zHRg), illustrating their opposing dynamics. Figure combines zFlow, zBarriers, and zSDg as dependent variables with zHRg as independent variable, and displays the four EASFE phenomenological zones: high flow, medium flow, low flow, and no-flow.

In [28], it is experimentally demonstrated that barriers, when treated as the dependent variable, trace a concave curve with respect to the group’s mean heart rate in the control groups (r = 0.67, df = 15, F = 5.39, Sig. = 0.02). Equation (2) is, in turn, symmetric with the convex curve that emerges when the group’s mean flow is taken as the dependent variable and the group’s mean heart rate as the independent variable (r = 0.68, df = 13, F = 4.84, Sig. = 0.03). Together, both curves form a shape reminiscent of the silhouette of a fish (Figure 8). Because of this, barriers act as the antithesis of flow, also coined as asperities [28].

In Figure 8, we also present the graphical relationships between flow and barriers, expressed as *z* values as described in [28], alongside the four phenomenological zones considered in EASFE (high flow, medium flow, low flow, and no-flow conditions). In this graph, to visualize multiple variables within two dimensions, the dependent variables—mean group flow (zFlow), mean barriers (zBarriers), and the standard deviation of the group’s mean heart rate (zSDg)—were combined, while mean heart rate (zHRg) was used as the independent variable. Phenomenological flow zones were experimentally defined with zFlow, zSDg and zHRg. Nevertheless, curves were drawn with zHRg, zFlow, and zBarriers, respectively.

Furthermore, ref. [28] reported a strong and significant relationship—overlapping with the zFlow curve in Figure 8—between mean group cognitive absorption (zD1) and the mean group heart rate (r = 0.92, df = 12, F = 25.55, Sig. < 0.001).

In Table 2, we present a summary of the results for the control groups. The table includes the group and session assessed, the mean group heart rate in beats per minute (HRg), the mean group heart rate standard deviation (SDg), and the mean group flow expressed as a percentage of the maximum value measurable with the EduFlow scale. Note that HRV values correspond to the physiological measures obtained in the first five-minute span, whereas students response flow questionnaires at the end of the sessions. Moreover, HRg and SDg represent low computational cost HRV parameters linked to the sympathetic and parasympathetic nervous systems [20] respectively, while D1g is a key element of the EduFlow scale [23].

In addition, Table 3 presents similar information for the experimental groups, together with the EASFE flow state classification, as defined in [28]. To ensure that both control and experimental groups followed the same scheduled activities, the teacher adhered to the same curriculum but repeated explanations as suggested by the EASFE system, without being prompted by students. Accordingly, the teacher repeated once (Medium Flow), repeated twice (Low Flow), or repeated twice after a break to calm students (No Flow). In ascending order of occurrence, expressed as a percentage of the EduFlow maximum value, the distribution was as follows: High Flow (0%), No Flow (6.25%), Low Flow (18.75%), and Medium Flow (75%). Thus, he always repeated explanations at least one time.

Once the teacher’s attitude was described, we measured HRg Spearman’s correlations and significance among control and experimental groups regarding SDg, barriers, EduFlow, challenge, skill, and balance between control and experimental groups (Table 4).

Finally, Table 5 examines how students’ perception of balance (A3) changes in the experimental and control groups. Students rated this item on a 9-point Likert scale. The table reports the mean perceived balance (A3) and the standard deviation for each group, ordered by session number. It should be noted that students in the N5_2 group did not participate in the last two sessions due to lack of access to Roblox Studio.

In addition, knowledge assessments were administered before and after students participated in the instructional designs. In the pre-test, no student demonstrated prior knowledge, as evidenced by practical tasks and basic multiple-choice questions, with all groups scoring 0 out of 10 possible points. In the post-test, the mean scores and standard deviations were as follows: N5_1 (7.03 ± 1.42), N5_2 (7.70 ± 1.11), N6_1(6.35 ± 1.47), and N6_2 (7.69 ± 1.78).

**Figure 9:**
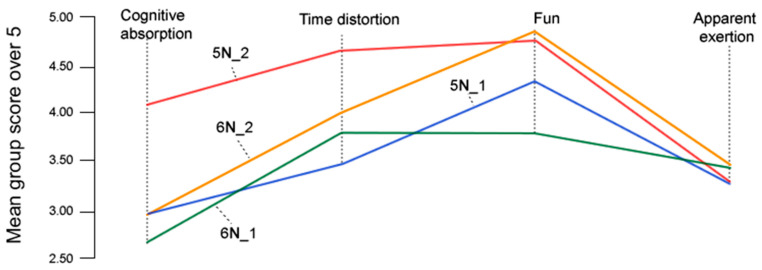
Line plots showing group mean scores for the following questions after interviews across four study groups: cognitive absorption (concentration), time distortion (sense of time flying), fun, and apparent exertion, each rated on a five-point scale.

Moreover, pupils rated the courses in a 0 to 10 scale, as follows: N5_1 (9.00), N5_2 (9.00), N6_1(9.04), and N6_2 (9.54). Additionally, participants were asked to rate on a five-point scale how concentrated they were (cognitive absorption), whether they experienced a sense of time flying (time distortion), whether they had fun (fun), and whether they had to exert substantial effort (apparent exertion). Figure 9 summarizes the group mean values for each item across the four study groups.

## 4. Discussion

First, we consider RQ1: What is the degree of coherence between the flow reported by the control groups and the flow recalled by the teacher using the EduFlow and FKS scales?

We observed coherent signals in the control groups and both FKS and EduFlow scales in the Bland–Altman plots (Figure 4, Figure 5, Figure 6 and Figure 7). Moreover, the experimental points are aligned around the balance line within the marked confidence intervals (95%), which is a standard confidence ratio. However, since the goodness of fit is arbitrarily chosen by the researcher based on the precision deemed suitable for the study, we calculated the Root Mean Square Error (RMSE) of the experimental points relative to the balance line. We considered the RMSE values small (3.64, 2.54, 5.29, and 3.05), given that the EduFlow and FKS scales allow maximum scores of 84 and 77 points, respectively [23,24]. That means errors of 4.7%, 3.3%, 6.3%, and 3.63%, respectively.

In cases where the balance lines are horizontal, and positioned below zero, even though the teacher reports less flow than the averaged pupils, there is high coherence between the signals (Figure 4 and Figure 6). Moreover, the greater effort made by the teacher may be observed in the monotony of the S1 and S2 HRV parameters computed in Kubios software reports for every lesson, which describes Poincaré plots where S1 is noticeably greater than S2, suggesting a significant activation of the SNS against the PNS (see [20,48]). Those observations combined show a high degree of coherence between the flow reported by the groups of students and the teacher.

To address RQ2 (Is there a differential correlation between control and experimental groups among HR, balance, and flow?), we focus on Figure 3. The frequency histogram suggests that students perceive almost perfect balance in the tasks in all groups, because the normality of the histogram distribution is disrupted by the prominent modal value of 5 (perfect balance). However, there is a meaningful difference, with large effect size, and statistical power in flow levels expressed as EduFlow scale among control (M = 61.95, SD = 6.42) and experimental groups (M =72.92, SD = 7.32), favouring experimental groups versus the control ones (t= −4.032, gl = 28, Sig. < 0.001, d = 1.480633, 1-β = 0.95, *α* = 0.01). It suggests enhanced wellness [1] in groups using the EASFE synchronous advice.

**Figure 10:**
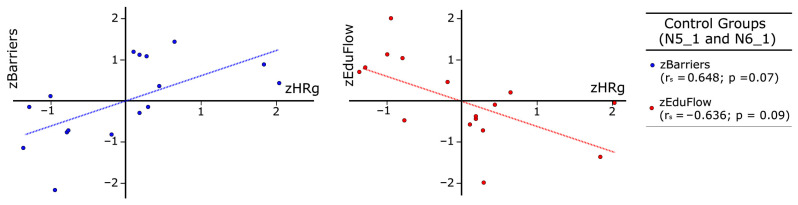
Relationship among zHRg, zBarriers, and zEduflow in control groups.

**Figure 11:**
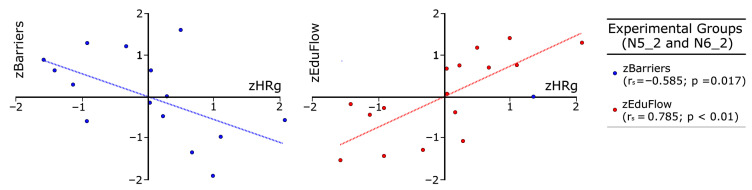
Relationship among zHRg, zBarriers, and zEduflow in experimental groups.

Next, in Figure 10 and Figure 11, we created several scatter plots with z-values for control and experimental groups respectively, using data from Table 3, Table 4 and Table 5. The x-axis represents HRg as the independent variable, while the y-axis shows both EduFlow and barriers as dependent variables. Such graphs allow us to compare meaningful group differences among barriers and flow with respect to heart rate. Blue points correspond to the group mean normalized barriers, while red points represent the mean normalized flow, measured using EduFlow questionnaires. Blue dotted lines show trends between barriers and heart rate, while red dotted lines indicate general trends between EduFlow and heart rate variables.

Figure 10 corresponds to the control groups, while Figure 11 represents the experimental groups. Note that we also drew dotted lines using least squares regression techniques to identify trends in the control and experimental groups for the readers, along with Spearman correlation analysis between the study variables for these groups to facilitate comparison.

Following Kuckartz et al. [49], p. 213, we discuss Spearman’s correlations in Figure 10 and Table 5. These show high and meaningful correlations among HR and dependent variables barriers and EduFlow (rs = 0.648, *p* = 0.007; rs = −0.626, *p* = 0.009; rs = −0.586, *p* = 0.017), but very high in experimental groups with EduFlow (rs = 0.785, *p* < 0.001). In contrast, correlations among HR, balance, or challenge are not significant in any group, which raises concerns about the axiom of balance, as these concepts are central to Flow Theory and may not be fully explained through physiological observations. This contrasts with barriers, a new concept discussed here. This may be influenced by the monotonous perceived balance (M = 5.10, SD = 1.14), but this is not the case for challenge.

Nevertheless, we found high, significant and mutually inverse correlations among HR and skills, when comparing the control group (rs = −0.611, *p* = 0.012) in Figure 8 with the experimental group (rs = 0.792, *p* < 0.001) in Figure 11. The correlation signs are also inversed among skills and barriers, addressing control or experimental groups, if HR is the independent variable (Table 5). In addition, barriers address the asperities recalled in activities in our context and has opposite correlation with flow trends in both groups, in coherence with flow phenomenology. Consequently, the impact of EASFE suggestions has both qualitative and quantitative effect on experimental groups physiology, because graphs in Figure 9 and Figure 10 exhibit mirror horizontal symmetry respect to HR, resembling in simple ways the sympathetic nervous system activation [20,48]. Moreover, correlation is not demonstrated between HR and SD in control groups (sr = 0.024, *p* = 0.931), in opposition with experimental groups (sr = 0.594, *p* = 0.015). Since SD reveals the antagonistic parasympathetic modulation [20,48], it may suggest a higher prevalence of calm in experimental groups, in which flow recall is higher. If we consider all these findings together, we can respond affirmatively to RQ2, regarding flow and HR correlation, but negatively for balance. Although meaningful alternative graphs are provided in Figure 11, this appears disconcerting, because the balance axiom is essential in Flow Theory and in the educational practice [1,3].

We will address RQ3 (What are the differences in perceived group balance between experimental and control groups as measured by Poincaré plots?) using Poincaré graphs (see [50]) to study the nonlinear behaviour of the balance dimension in the FKS scales among control and experimental groups. They are scatter plots in which each measure of a time series for a time t is correlated with the value at the subsequent time (t + 1). We also represent flow lines by joining the experimental points with vectors.

**Figure 12:**
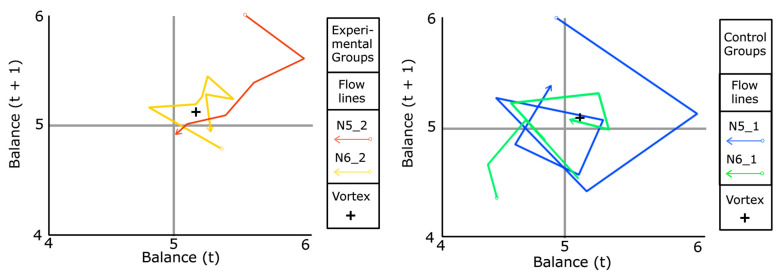
Poincaré plots for average variable A3 per session in experimental groups (**left**) and control groups (**right**).

In Figure 12, we create Poincaré plots with the data from Table 6 (average balance per session of the groups). Four flow lines appear to be drawn toward a vortex, slightly above the perfect equilibrium point (x = 5.0, y = 5.0). We show a closer detail of the vorticity zone, separating the experimental groups (N5_2 and N6_2) from the control groups (N5_1 and N6_1). Additionally, we mark the average value of variable A3 (balance) in both graphs with a black cross (M = 5.11, SD = 0.39). This corresponds to the place where the centre of an attractor vortex is inferred to be, slightly shifted upwards and to the right, relative to the perfect equilibrium point (coordinates x = 5.0, y = 5.0). We will specify the hypothetical position of the vortex later.

The flow lines in Figure 12 appear consistent with the postulates of the mathematical model for individual flow proposed by Melnikoff, Carlson, and Stillman [51]. This model involves concepts of Shannon’s information entropy [52] and intelligent agents [53]. We assumed that the teacher acts as an intelligent agent, occasionally assisted by quality information, provided by a computational system (see [52,54]). In this regard, we observed that the flow curves in the Poincaré plots for the group balance variable erratically move toward the attractor vortex.

Next, we calculated Shannon’s entropy (H) according to Equation (3), where $p_{i}$ is the probability of the i-th score on the Likert scale (9 points).

(3) $$H=-\sum_{i=1}^{9}p_{l}\log_{2}\left(p_{i}\right)$$

To determine $p_{i}$, we used Equation (4), which requires $n_{i}$ (the number of times the score i was selected) and the total number of responses (N).

(4) $$p_{i}=\frac{n_{i}}{N}$$

These calculations were performed with Python 3.10 using the data from Table 6. They are shared and clarified with the help of Google Colab (see Supplementary Materials). The average results of H for each participating group are presented in Table 6, which does not include the missing data from sessions 8 and 9 in the design groups, to make them comparable with each other.

In group N5_1, the session with the lowest H is the first one, having a perceived balance of A3 = 4.92 ± 0.52 (see Table 6). In group N5_2, the minimum is found in session 6 (A3 = 5.00 ± 0.43). Group N6_1 has the lowest H in session 4 (A3 = 5.07 ± 0.73). In contrast, in the fourth session of the N6_1 group the minimum H is in (A3 = 5.20 ± 0.41). Likewise, in the last row of Table 6, we have the average entropy of each group ($\bar{H}$), discounting the sessions of Roblox Studio (8 and 9 in experimental groups). It is observed that the average entropy is higher in the graphic design control group ($\bar{H}$ = 2.29) than in its experimental counterpart ($\bar{H}$ = 1.94). The same happens between the control group of robotics ($\bar{H}$ = 2.31) and the experimental group ($\bar{H}$ = 1.70).

**Figure 13:**
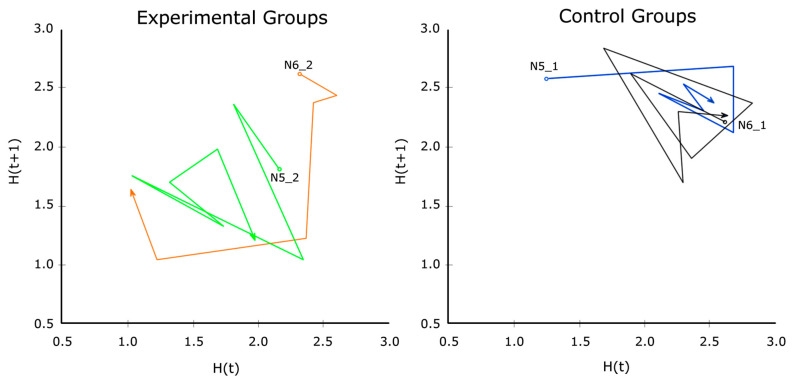
Poincaré plot for Shannon’s entropy (H) in study groups.

To investigate the nonlinear evolution of H in all groups, we created the corresponding Poincaré plot. Figure 13 outlines flow lines that swirl between the values 2 and 2.5 for H (t + 1) in the control groups. Both end up at the value H (t) = 2, pointing toward 3. In contrast, the flow lines of the experimental groups move toward H (t + 1) values below 2 and end up near position 1.5. The lowest Shannon entropy values correspond to the experimental groups, in contrast to those of the control groups. Therefore, in the experimental groups, Shannon entropy is lower, which indicates greater efficiency. This suggests that the advisory system provides effective mechanisms for supporting teachers in making relevant and informed decisions.

Additionally, the value with the lowest H for the Balance variable (in Table 6, 1.04) corresponds to session 4 of group N6_2 (A3 = 5.20 ± 0.414). On the Likert scale, this is slightly above perfect balance (5), which seems consistent with the position of a hypothetical attractor vortex in Figure 13, whose position could be further adjusted if we had more data.

Likewise, following Vygotsky [55,56] redefined the Zone of Proximal Development as a 45-degree inclined strip parallel to the flow zone in the channel model, just below the anxiety zone. Pedagogically, the model illustrates that, through non-resolutive sequential aids and training, the teacher facilitates the acquisition of knowledge and skills that exceed the isolated capacities of the learner. Additionally, the teacher can approach this ZPD by making tasks more difficult or handicapping students when tasks become too easy [1]. Precisely, the attractor vortex is conceptually located in the ZPD for Flow Theory [56], for the averages of the balance variable for each group of students.

In summary, from Figure 12 and Figure 13 we see that in the EASFE-assisted groups, the flow lines head more directly toward the hypothetical location of the attractor vortex, meaning to the PDZ, represented by the average value of the variable A3 (balance perceived) across all studied groups (M = 5.11, SD = 0.39), or orbit closer to it. However, flow lines in the control group are more erratic, with trajectories spreading out more around the vortex and yielding higher H values.

Additionally, the results indicate that the experimental groups recalled higher levels of cognitive absorption, time distortion, and fun than the control groups, while reporting similar levels of exertion (Figure 9). This is coherent with the similar location in all groups of the attractor vortex (same ZPD in Figure 12), but higher mean group flow levels after sessions reported in experimental groups with EduFlow questionnaires (t = −4.03, gl = 28, Sig. < 0.001, d = 1.48, 1-β = 0.95, *α* = 0.01). Similarly, satisfaction with the courses was high, scoring at least 9 out of 10 points in all groups, and slightly higher in experimental groups.

If we consider findings from RQ1, RQ2, and RQ3 together, with a preliminary scope, we suggest that:

(1) Although teachers usually try to bring their students to the ZPD by trial and error in metacognitive terms of flow, students can reach that ZPD more straightforward when the teacher guides them using a physiological-measures-based tool. This is consistent with the phenomenology observed in a classroom and with the balance axiom in Flow Theory [1,3].
(2) We quantify the contradiction noted by [10] between measured flow and balance (r = 0.206, Sig. < 0.01), suggesting that being a volatile phenomenon, it may be more suitable for nonlinear analysis techniques in group contexts, which offer average values.
(3) The “attractor vortex hypothesis” can be posed stating that teacher’s task is guiding students to the ZPD by considering the nonlinear patterns of group flow rather than looking for the perfect balance between challenge and skills.

## 5. Conclusions, Limitations, and Further Research

In this paper we addressed difficulties in the definition and the importance of the Axiom of Flow in natural educational contexts, using a two-academic-courses experience in which we physiologically assessed two control and two experimental groups. Surprising results obtained from this study arose from the use of no lineal analysis techniques to assess such a volatile state instead of lineal and recall-based techniques.

In response to RQ1, we conclude that there is a clear coherence among the teacher’s and student’s group flow recalled, despite performing different tasks for eight one-hour sessions. Therefore, we propose as a hypothesis for future research that the teacher evaluates the groups based on flow metacognitions (see [7,27]). This gives a measure of the importance of Flow Theory phenomenology in educational settings. Since we had only one teacher, we understand that he possesses good empathy, requiring more teachers to generalize these observations. Conversely, the teacher was invariant but the help of the EASFE as an external tool provided him with additional information to guide his actions during lessons. Future studies involving a specialized group of teachers would help to improve learning designs and methods, and to examine potential differences in empathetic sensitivity toward student groups. Moreover, the teacher also wore a heart rate sensor, which provides evidence of the very high cognitive—and even physical—demands that iSTEAM methodologies may impose to be effective. Including multiple teachers would help determine whether this is a general tendency, which could, in turn, hinder the broader and more effective adoption of these active methodologies.

Regarding RQ2, we found differential correlation patterns between control and experimental groups among HR and flow, but not with the balance. Correlation patterns among HRV parameters assessed (HR and SD), barriers, and flow are coherent with flow phenomenology. Moreover, we found that context plays a prominent role since scenarios with EASFE resulted more calming (experimental groups) due to a guided behaviour of the teacher, in line with students’ needs. In contrast, in the control groups, the teacher only provided aid upon students’ request, which may create a more stressful context. Hence, phenomenological zones in channel flow models may be more efficient if they refer to groups of people and contexts rather than to individual psychological states and should only be considered conceptually. Furthermore, this paper sheds light on the disparity of correlations accounted among HR and the physiology of flow observed in systematic reviews (i.e., [17,18]). We found negative, positive, and insignificant correlations in the same work, strongly dependent on the context.

Considering RQ3, the subjective balance of tasks appears to be crucial, although Løvoll and Vittersø [16] have suggested that monotony in balance can lead to boredom. However, nonlinear processes with significance across sessions must be considered. Even though that balance is not entirely perfect we think that it is advisable, especially in educational practice. Furthermore, regarding the balance variable, we posit the “attractor vortex hypothesis” which explains that these nonlinear differences could be attributed to the guided actions of the teacher who can now adjust his efforts to the actual needs of students instead of doing it through trial and error in metacognitive terms of flow, as he made in control not guided groups. By doing so, the teacher can optimally guide students toward the ZPD for flow [56], as suggested by flow lines and their vorticity (Figure 12). This is consistent with the phenomenology observed in a classroom and with the balance axiom in Flow Theory [1,3]. Furthermore, it addresses the contradiction found by [10] between measured flow and balance (r = 0.206, Sig. < 0.01), suggesting that due to its volatile nature, nonlinear analysis techniques in group contexts may be more suitable, offering average values.

Regarding the insightful mathematical model offered by Melnikoff, Carlson, and Stillman [51] on Flow Theory for individuals, we want to highlight several points for future work. Firstly, our data show significant results based on probabilistic projections and physiological measurements in just 5 min of teaching time, whereas real sessions can span up to an hour. These sessions involve a variety of cognitively complex activities. None of the students dropped out of the courses, despite being free to do so, while levels of flow prevalence and physiological measures varied. Therefore, we assume that reported flow is mediated by expectations generated to experience it physiologically, which are formed at the beginning of classes [28]. It may also be relevant in educational practice, as it is not uncommon to reward students with more rewarding activities at the end of sessions rather than at the beginning. Additionally, it seems feasible to evaluate the suitability of instructional designs and teaching quality through EASFE.

Regarding the consistently high stress levels revealed by physiology, it suggests reconsidering the strain that active methodologies may impose on teachers. It also highlights the energy expenditure undertaken by students derived from HRV parameters, which could be calculated in subsequent studies on ergonomics and occupational health.

Moreover, Swann et al. [4] have pointed out that Flow Theory is at a critical juncture, considering a paradigm shift or a more precise definition of the concept. While this paper revises the axiom of balance, further work remains to be done. We advocate for phenomenological approaches to address the complexity of reality while leveraging precise quantitative research methods.

Among the limitations, it should be noted that only one teacher was involved across all research groups; therefore, future work should consider the inclusion of multiple lecturers. Additionally, the quasi-experimental design, intended to address the ecological conditions of real-world contexts, may introduce biases in sample selection. In this regard, the participants were drawn from four relatively small, authentic classroom groups within the same primary school, consisting of students approaching secondary school age. Importantly, the natural structure and dynamics of these classes could not be disrupted. Constraints of space, time, ethics, and economics prevent us from including more groups and sessions, given the complexity of the STEAM methodology used compared to other possibilities. Only two themes have been employed (design and robotics) in this regard.

## Figures and Tables

**Table 1:** Sample characteristics of groups.

Group	N	Girls	Boys	Age (Mean ± SD)
Control 5N_1	14	7	7	10.80 ± 0.44
Control 6N_1	14	6	8	11.82 ± 0.31
Experimental 5N_2	14	7	7	10.56 ± 0.34
Experimental 6N_2	14	9	5	11.46 ± 0.53

**Table 2:** Comparison of mean HRV parameters (HR, SD) and flow scale scores (barriers, EduFlow) across control groups after first five minutes (Span 1).

Group	HRg	SDg	Barriers	EduFlow (%)
N5_1_2	77.82	6.47	−0.71	58.81
N5_1_3	98.03	6.86	−0.40	61.69
N5_1_4	85.49	6.13	−0.56	49.11
N5_1_5	96.55	6.22	−0.29	53.06
N5_1_6	84.69	7.04	−0.60	59.14
N5_1_7	88.02	6.92	−0.14	62.99
N5_1_8	84.18	6.93	−0.2	58.12
N5_1_9	85.37	6.99	−0.23	57.14
N6_1_2	76.55	6.53	−1.09	74.66
N6_1_3	74.10	6.74	−0.56	67.06
N6_1_4	76.20	7.18	−0.49	69.05
N6_1_5	77.71	6.29	−0.72	68.66
N6_1_6	73.67	6.50	−0.82	66.37
N6_1_7	84.76	6.52	−0.22	59.38
N6_1_8	81.99	6.48	−0.73	64.81
N6_1_9	86.56	7.11	−0.43	61.11

**Table 3:** Comparison of mean HRV parameters (HR, SD) and flow scale scores (barriers, EduFlow) across experimental groups after the first five minutes (Span 1), with EASFE flow level recommendations included.

Group	HRg	SDg	Barriers	D1g	EduFlow (%)	EASFE Flow Level
5N2_1	94.33	7.33	−0.90	10.83	83.23	Low
5N2_2	90.99	6.94	−0.64	12.64	78.36	Medium
5N2_3	92.17	7.55	−0.26	10.40	81.61	Medium
5N2_4	99.00	8.38	−0.66	11.63	82.23	Low
5N2_5	94.75	6.05	−0.73	12.67	78.48	No Flow
5N2_6	92.90	7.07	−0.80	13.82	77.88	Low
5N2_7	90.12	6.32	−0.58	13.36	77.78	Medium
6N2_1	86.02	4.90	−0.66	10.50	70.79	Medium
6N2_2	83.82	5.76	−0.43	16.43	71.51	Medium
6N2_3	90.18	6.78	−0.43	16.33	73.26	Medium
6N2_4	85.10	6.94	−0.50	14.70	69.56	Medium
6N2_5	90.72	5.13	−0.62	15.50	70.14	Medium
6N2_6	83.12	5.34	−0.39	16.00	61.51	Medium
6N2_7	85.99	6.52	−0.31	13.56	62.18	Medium
6N2_8	88.56	6.20	−0.33	14.78	63.31	Medium
6N2_9	91.23	8.07	−0.55	13.67	64.92	Medium

**Table 4:** HRg Spearman’s correlations among control and experimental groups (n = 16), including HRg standard deviation, barriers, EduFlow, challenge, skills, and balance scores.

		SDg	Barriers	EduFlow	Challenge	Skill	Balance
Correlation with HRg in Control Groups	Coef.	0.024	0.648	−0.626	0.328	−0.611	0.315
	Sig.	0.931	0.007	0.009	0.215	0.012	0.235
Correlation with HRg in Experimental Groups	Coef.	0.594	−0.586	0.785	−0.157	0.792	0.185
	Sig.	0.015	0.017	<0.001	0.560	<0.001	0.492

**Table 5:** Comparative of perceived mean balance (A3) among control (N5_1, N6_1) and experimental groups (N5_2, N6_2) by sessions.

	N5_1 (Design)		N5_2 (Design)		N6_1 (Robotics)		N6_2 (Robotics)	
Session	Mean	SD	Mean	SD	Mean	SD	Mean	SD
1	4.92	0.515	5.55	1.214	4.43	1.512	5.33	1.047
2	6.00	1.732	6.00	1.789	4.36	1.120	4.79	0.802
3	5.14	1.657	5.60	1.350	4.67	1.155	5.15	1.214
4	4.43	1.618	5.38	1.261	5.07	0.730	5.20	0.414
5	5.27	1.009	5.08	0.494	4.55	1.968	5.25	0.754
6	5.08	1.320	5.00	0.426	5.23	1.235	5.44	0.527
7	4.57	1.158	4.92	0.669	5.31	0.855	5.23	0.725
8	4.86	1.406			5.00	1.537	5.27	0.905
9	5.38	1.261			5.08	1.084	4.92	0.494

**Table 6:** Shannon’s entropy (H) and its average in study groups by sessions.

Session	H for N5_1 (Design)	H for N5_2 (Design)	H for N6_1 (Robotics)	H for N6_2 (Robotics)
1	1.28	2.32	2.61	2.16
2	2.58	2.60	2.25	1.82
3	2.67	2.42	2.29	2.34
4	2.67	2.37	1.70	1.04
5	2.11	1.23	2.82	1.74
6	2.44	1.04	2.36	1.32
7	2.30	1.59	1.90	1.69
8	2.53		2.61	1.97
9	2.37		2.21	1.23
Mean H (n)	2.29 (n = 7)	1.94 (n = 7)	2.31 (n = 9)	1.70 (n = 9)

## Data Availability

The datasets are not publicly available for ethical reasons, as they include sensitive biometric information. Aggregated values at the study-group level are provided instead in the tables of this paper. Further inquiries can be directed to the corresponding author.

## Supplementary Materials

The following supporting information can be downloaded at: https://www.mdpi.com/article/10.3390/s26010038/s1, or https://acortar.link/p5DZcU (accessed on 20 October 2025).

## Abbreviations

The following abbreviations are used in this manuscript:

A1	Subjective Challenge (FKS; 9-point Likert)
A2	Subjective Skills (FKS; 9-point Likert)
A3	Balance (FKS; 9-point Likert)
API	Application Programming Interface
BA-plotteR	Web tool for generating Bland–Altman plots and limits of agreement
Barriers	Nonlinear index of perceived task asperities: Barriers = (Ā1 − Ā2)/Ā3
CSV	Comma-Separated Values
D1–D4 (EduFlow)	D1: Cognitive Absorption; D2: Time Transformation; D3: Loss of Self-Consciousness; D4: Autotelic Experience/Well-Being (7-point Likert each)
D1g	Group mean score of EduFlow D1 (Cognitive Absorption)
EASFE	Early Alert System of Flow Expectation (synchronous classroom advisory system)
EFM	Experience Fluctuation Model
EduFlow	Flow scale for learning contexts (four dimensions D1–D4)
FKS	Flow Kurz Skala/Flow Short Scale (includes A1–A3)
FMQ	Flow Metacognitions Questionnaire
H	Shannon’s entropy (bits)
$\bar{H}$	Mean Shannon’s entropy (bits)
HR	Heart Rate (beats per minute, bpm)
HRg	Group mean heart rate over a defined span (bpm)
HRV	Heart Rate Variability
ICT	Information and Communication Technology
JSON	JavaScript Object Notation
PCA	Principal Component Analysis
PNS	Parasympathetic Nervous System
REST	Representational State Transfer (web service style)
RMSE	Root Mean Square Error (relative to Bland–Altman balance line)
S1/S2 (Poincaré)	Short-term (SD1)/long-term (SD2) HRV descriptors from Poincaré plots
SCORM	Sharable Content Object Reference Model
SD	Standard Deviation
SDg	Group standard deviation of HR over a defined span (bpm)
SDK	Software Development Kit
SNS	Sympathetic Nervous System
Span	Five-minute analysis window (e.g., Span 1 = first 5 min of a lesson)
STEAM	Integrated Science-Technology-Engineering-Arts-Mathematics methodology
STEM	Science-Technology-Engineering-Mathematics subjects
ZPD	Zone of Proximal Development
5N_1/6N_1	Control groups (Grade 5/Grade 6)
5N_2/6N_2	Experimental groups (Grade 5/Grade 6)
subscript “g”	Denotes group mean (e.g., HRg, SDg, D1g)
